# Distinct role of subunits of the *Arabidopsis* RNA polymerase II elongation factor PAF1C in transcriptional reprogramming

**DOI:** 10.3389/fpls.2022.974625

**Published:** 2022-09-29

**Authors:** Simon Obermeyer, Richard Stöckl, Tobias Schnekenburger, Christoph Moehle, Uwe Schwartz, Klaus D. Grasser

**Affiliations:** ^1^ Cell Biology & Plant Biochemistry, Biochemistry Centre, University of Regensburg, Regensburg, Germany; ^2^ Center of Excellence for Fluorescent Bioanalytics (KFB), University of Regensburg, Regensburg, Germany; ^3^ NGS Analysis Centre, Biology and Pre-Clinical Medicine, University of Regensburg, Regensburg, Germany

**Keywords:** *Arabidopsis thaliana*, chromatin, histone modifications, PAF1C, RNA polymerase II, transcript elongation

## Abstract

Transcript elongation by RNA polymerase II (RNAPII) is dynamic and highly regulated, thereby contributing to the implementation of gene expression programs during plant development or in response to environmental cues. The heterohexameric polymerase-associated factor 1 complex (PAF1C) stabilizes the RNAPII elongation complex promoting efficient transcript synthesis. In addition, PAF1C links transcriptional elongation with various post-translational histone modifications at transcribed loci. We have exposed *Arabidopsis* mutants deficient in the PAF1C subunits ELF7 or CDC73 to elevated NaCl concentrations to provoke a transcriptional response. The growth of *elf7* plants was reduced relative to that of wildtype under these challenging conditions, whereas *cdc73* plants exhibited rather enhanced tolerance. Profiling of the transcriptional changes upon NaCl exposure revealed that *cdc73* responded similar to wildtype. Relative to wildtype and *cdc73*, the transcriptional response of *elf7* plants was severely reduced in accord with their greater susceptibility to NaCl. The data also imply that CDC73 is more relevant for the transcription of longer genes. Despite the fact that both ELF7 and CDC73 are part of PAF1C the strikingly different transcriptional response of the mutants upon NaCl exposure suggests that the subunits have (partially) specific functions.

## Introduction

In eukaryotic cells, hundreds of proteins regulate the transcription of genes by RNA polymerase II (RNAPII). The majority of these proteins control transcriptional initiation *via* interaction with promoter regions. Functionally distinct so-called transcript elongation factors (TEFs) modulate the efficiency of mRNA synthesis by different mechanisms after the initiation stage. Some TEFs act as histone modifiers, histone chaperones or chromatin remodelers, manipulating properties of the nucleosomal template, whereas others adjust the catalytic activity of RNAPII directly (Sims et al., 2004; Kwak and Lis, 2013; Chen et al., 2018). A remarkable example of a regulator of transcriptional elongation by RNAPII is the multifunctional polymerase-associated factor 1 complex (PAF1C) that originally was discovered and characterized in *Saccharomyces cerevisiae* (Wade et al., 1996; Mueller and Jaehning, 2002). Yeast PAF1C is composed of five subunits Paf1, Ctr9, Leo1, Rtf1 and Cdc73 and it interacts directly with elongating RNAPII (Jaehning, 2010). In mammals, PAF1C contains an additional subunit WDR61 (Ski8), while RTF1 appears to be less stably associated (Francette et al., 2021). Recent cryo-electron microscopy studies revealed detailed structural insight into the architecture and molecular interactions of elongation competent RNAPII associated with PAF1C and other TEFs (Vos et al., 2018; Vos et al., 2020). The structural information suggests that association of PAF1C with RNAPII allosterically contributes to the stabilization of the elongation complex, which is in line with its stimulation of productive transcript synthesis (Rondón et al., 2004; Hou et al., 2019). Moreover, PAF1C links transcript elongation with various post-translational histone modifications over transcribed regions, including H2B mono-ubiquitination (Wood et al., 2003; Xiao et al., 2005), H3K4me2/3 and H3K79me2/3 (Krogan et al., 2003; Ng et al., 2003; Wood et al., 2003), as well as H3K36me3 (Chu et al., 2007). Thus, PAF1C acts at the interface of transcription and chromatin, modulating the progression of RNAPII during elongation.

In plants, PAF1C is conserved and was initially recognized because of its role in the transition from vegetative to reproductive development (van Lijsebettens and Grasser, 2014). The PAF1C subunits were named after the *Arabidopsis* mutants, whose analysis for their developmental phenotype led to identification of the genes encoding the respective subunits (He et al., 2004; Oh et al., 2004). Accordingly, most of the subunits based on the mutants were termed early flowering (*elf*) and/or vernalization independence (*vip*): PAF1 (At-ELF7), CTR9 (At-ELF8, At-VIP6), LEO1 (At-VIP4), RTF1 (At-VIP5), WDR61/SKI8 (At-VIP3) and CDC73 (At-CDC73, At-PHP) (He et al., 2004; Oh et al., 2004; Park et al., 2010). Co-immunoprecipitation and affinity-purification in combination with mass spectrometry analyses revealed that in common with mammals, *Arabidopsis* PAF1C consists of these six subunits (Oh et al., 2004; Antosz et al., 2017). *Arabidopsis* mutants deficient in PAF1C subunits VIP3, VIP4, VIP5, VIP6/ELF8 and ELF7 exhibit marked early flowering phenotypes that are commonly associated by reduced expression of the floral repressor *FLC* (and paralogs) (Zhang and van Nocker, 2002; Zhang et al., 2003; He et al., 2004; Oh et al., 2004). Moreover, these mutants are significantly smaller than wild type and often exhibit defects in inflorescence and flower morphology (Zhang and van Nocker, 2002; Zhang et al., 2003; He et al., 2004; Fal et al., 2017). Mutants defective in the CDC73/PHP subunit share the early flowering phenotype (and reduced *FLC* expression), but are of wild type size and display normal flower development (Park et al., 2010; Yu and Michaels, 2010). The decreased expression of *FLC* in mutants lacking PAF1C subunits is likely mediated by altered levels and distribution of histone H3 methylation marks at the *FLC* locus (e.g. H3K4me3, H3K27me3, H3K36me2) (He et al., 2004; Oh et al., 2008; Xu et al., 2008; Park et al., 2010; Yu and Michaels, 2010). PAF1C also modulates the temperature-responsive transition to flowering (Nasim et al., 2022). Beyond that PAF1C is required for plant response to repeated touch stimuli, as *Arabidopsis* mutants deficient in VIP3, VIP5 and VIP6 did not react to the mechanical stimulation as the wild type. VIP3 proved necessary for the touch-induced upregulation of the *TCH3* and *TCH4* mRNAs, a characteristic feature of the response to mechanical stimulation (Jensen et al., 2017).

The diversity of phenotypes associated with deficiency of PAF1C subunits in various organisms can be attributed to the misexpression of target genes and downstream processes. In yeast and mammals, there is accumulating evidence that inactivation of different PAF1C subunit genes to some extent causes distinct phenotypes, arguing for subunit specificity (Francette et al., 2021). This aspect has not been addressed in plants under transcriptional challenging conditions. Since chromatin-based reprogramming in reaction to environmental cues is crucial for plant growth (Kim, 2021), we studied here the transcriptional response of *Arabidopsis* PAF1C subunit mutants upon exposure to elevated NaCl concentrations.

## Materials and methods

### Plant cultivation and documentation

Seeds of *Arabidopsis thaliana* Col-0 were sown on solid 0.5x MS medium (Murashige and Skoog, 1962) and after stratification for 48h at 4°C in the dark, the plates were transferred to a plant incubator (PolyKlima) with long day settings (16h light (110 µmol·m^-2^·s^-1^) at 21°C and 8h darkness at 18°C. In some experiments the 0.5x MS medium was supplemented with 50 or 100 mM NaCl and for root growth assays plants were grown on vertically oriented 0.5x MS plates. Plant phenotypes including root growth were documented as previously described (Dürr et al., 2014; Antosz et al., 2020). Seeds of T-DNA insertion lines were obtained from the Arabidopsis stock centre (http://arabidopsis.info/): *elf7-2*, *elf7-3, elf8-1* (He et al., 2004); *cdc73-1*, *cdc73-2* (Park et al., 2010; Yu and Michaels, 2010) and GK_270G12 harboring a T-DNA insertion in exon 14 of *ELF8* (At2g06210) (Kleinboelting et al., 2012) termed *elf8-4*. Plant genotypes were examined by PCR analysis of genomic DNA isolated from leaves as previously described (Dürr et al., 2014; Antosz et al., 2020) using gene- and insertion-specific primers (**Table S1**).

### Isolation of RNA and cDNA synthesis

Nuclei were isolated from aerial parts of 7 days after stratification (DAS) plants as previously described (Pfab et al., 2017) and analyzed by fluorescence microscopy and immunoblotting as previously described (Antosz et al., 2017; Pfab et al., 2018). RNA was extracted from frozen nuclei using the TRIzol method (Invitrogen). After DNase treatment reverse transcription was performed using 1.5 µg of RNA, random hexameric primers and 200 U Reverse Transcriptase (Thermo Fisher Scientific) as previously described (Pfaff et al., 2018).

### RT-qPCR analyses

Amplification with cDNA as a template was performed using the Kapa SYBR FAST system (ThermoFisher Scientific) and gene-specific primers (**Table S1**) with a Mastercycler ep realplex 2 (Eppendorf) as previously described (Antosz et al., 2017). The qPCR measurements were analyzed using the ΔΔCt method implemented with the ‘pcr’ package (Ahmed and Kim, 2018).

### Transcript profiling by RNA-seq

Nuclear RNA isolated using the TRIzol method (Invitrogen) was further purified and DNase-treated using the Monarch RNA Cleanup Kit (New England Biolabs). Library preparation and RNA-seq were performed at the Genomics Core Facility (University of Regensburg, www.kfb-regensburg.de), employing the following modules: NuGEN Universal Plus RNA-Seq with NuQuant User Guide v3 (Tecan Genomics) in combination with Arabidopsis rRNA AnyDeplete module, the Illumina NextSeq 2000 System (Illumina), and the KAPA Library Quantification Kit-Illumina/ABI Prism (Roche Sequencing Solutions). To judge final library complexities (*vs*. PCR duplicates) unique molecular tags were used (Smith et al., 2017). Equimolar amounts of each library were sequenced on an Illumina NextSeq 2000 instrument controlled by the NextSeq 2000 Control Software (NCS, v1.4.0.39521), using 50 cycles P3 Flow Cell with the dual index, paired-end run parameters. Image analysis and base calling were done by the Real Time Analysis Software (RTA, v3.9.2). The resulting ‘.cbcl’ files were converted into ‘.fastq’ files with the bcl2fastq (v2.20) software.

### RNA-seq data analysis

Quality control was performed using fastQC (v0.11.9) and multiQC (v1.11) (Ewels et al., 2016). After the initial quality assessment, the molecular tag data was recorded in the header for each read *via* umi_tools (v1.1) (Smith et al., 2017). Reads with low base quality and adapter contaminations were removed using trimmomatic (v0.39, ‘ILLUMINACLIP : NGS_contaminants.fa:2:30:10 LEADING:3 TRAILING:3 SLIDINGWINDOW:4:15 MINLEN:36’) (Bolger et al., 2014). The remaining reads were mapped to the TAIR10 genome (Lamesch et al., 2012) using STAR (v2.7.9a, ‘–outFilterType BySJout –outFilterMultimapNmax 20 –alignSJoverhangMin 8 –alignSJDBoverhangMin 1

–outFilterMismatchNmax 999 –alignIntronMin 10 –alignIntronMax 1000000

–outFilterMismatchNoverReadLmax 0.04 –outSAMmultNmax 1 –outMultimapperOrder Random’). The resulting ‘.bam’ files were filtered to only include alignments with MAPQ score ≥ 10, sorted, and indexed using samtools (v1.3) (Danecek et al., 2021). Finally, the molecular tag data was used in conjunction with the alignments to remove technical duplicates using umi_tools (v1.1).

For the differential gene expression analysis, the resulting files from the pipeline outlined above were used to create a count table using the featureCounts function of the rsubread package (v2.4.3) (Liao et al., 2019), which was then analyzed using DESeq2 (v1.30.1) (Love et al., 2014) and the tidybulk package (v1.2.1) (Mangiola et al., 2021).

Differential splicing analysis was performed using DEXSeq v1.40 (Anders et al., 2012; Reyes et al., 2013). First, the exon annotation was prepared using the provided python script dexseq_prepare_annotation.py with the option ‘-r no’. Next, the read coverage of each exon was calculated using the dexseq_count.py script. Finally, salt stress-induced alternative splicing of either Col-0 or *cdc73-2* samples was tested using default settings and an FDR of 0.05.

### ChIP sequencing

Chromatin immunoprecipitation (ChIP) was essentially performed as previously described (Antosz et al., 2020; Michl-Holzinger et al., 2022). Plants (14-DAS *in vitro* grown) were crosslinked with formaldehyde and used for isolation of nuclei, before chromatin was sheared using a Bioruptor pico device (Diagenode). Immunoprecipitation was performed using antibodies directed against RNAPII-S2P (abcam, ab5095). For ChIP-seq, libraries were generated using NEBNext Ultra II DNA Library Prep Kit for Illumina (New England BioLabs) and the final libraries (3 replicates each) were sequenced by the Genomics Core Facility at the University of Regensburg using NextSeq 2000 (Illumina). Reads were aligned to the TAIR10 genome (https://www.arabidopsis.org/) using Bowtie2 (Langmead and Salzberg, 2012) and coverage tracks were calculated with deeptools “bamCoverage”. Downstream analysis was mainly performed using the deepTools2 suite (version 3.5.0) (Ramírez et al., 2016) and quality control was performed at several steps using FastQC (Ewels et al., 2016). Reads were mapped against the TAIR10 genome and regions with aberrant coverage or low sequence complexity were removed (Quadrana et al., 2016). After confirming high pairwise correlations, the biological replicates were merged and CPM normalized.

## Results

### Various PAF1C subunit mutants respond differently when exposed to elevated NaCl concentrations

**Figure 1:**
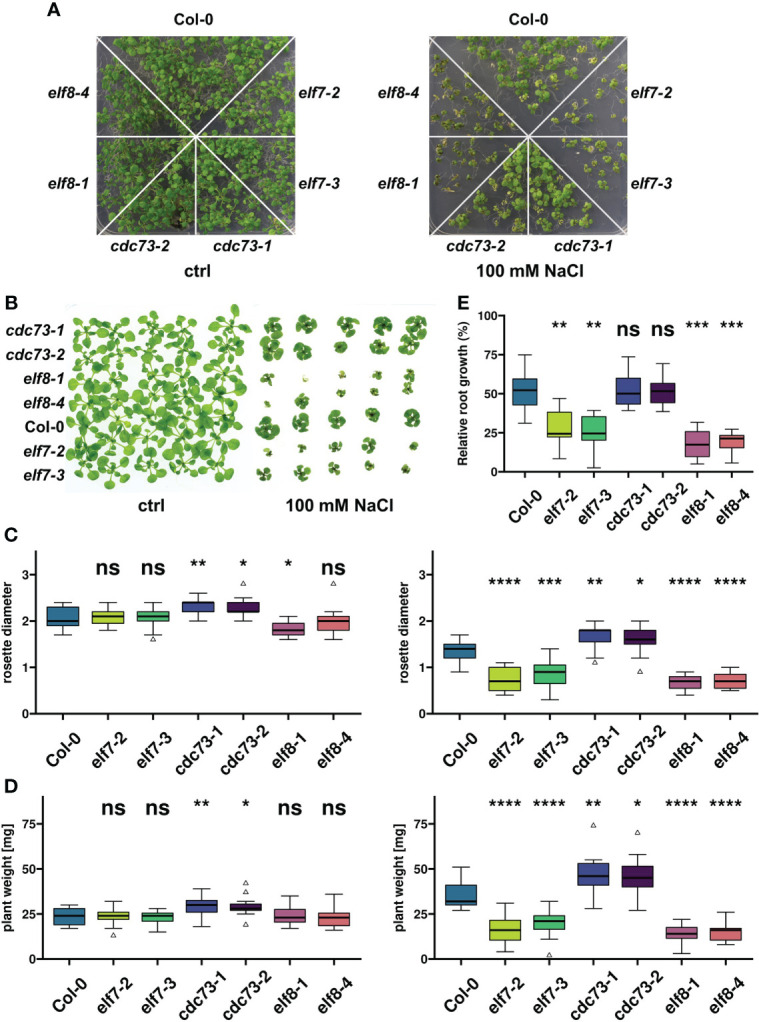
Growth of PAF1C subunit mutants is differentially affected by exposure to elevated NaCl concentrations. **(A)** Seeds of the indicated genotypes were sown on solid MS medium containing 100 mM NaCl or control medium (ctrl), and the resulting plants were documented at 14 days after stratification (14 DAS). **(B)** After growth on solid MS medium for 7 DAS, plants of the indicated genotypes were transferred to control medium or to medium containing 100 mM NaCl. Plants were documented after additional 18d (ctrl) or 21d (100 mM NaCl) of growth. **(C, D)** 7 DAS plants were grown in absence or presence of 100 mM NaCl for another 11d (ctrl, left) or 14d (100 mM NaCl, right), before rosette diameter [cm] **(C)** or fresh weight [mg] **(D)** was determined (n = 15). **(E)** Plants were grown on vertical MS plates for 7 DAS, before they were transferred to control medium or medium containing 100 mM NaCl. Length of the primary roots was recorded directly after transfer (0h) and 96h after transfer (n = 8). Growth after 96h in presence of NaCl is depicted as percentage of growth of the same genotype on control medium. The relative growth of each genotype was compared to Col-0 using Wilcoxon signed-rank test (ns, not significant; *, *p* ≤ 0.05; **, *p* ≤ 0.01; ***, *p* ≤ 0.001; ****, *p* ≤ 0.0001).

Based on preliminary tests with different environmental stress conditions we focused on elevated NaCl concentrations to provoke transcriptional reprogramming in PAF1C subunit mutants. Initially, seeds of the T-DNA insertion lines *elf7-2*, *elf7-3*, *elf8-1, elf8-4, cdc73-1*, *cdc73-2* and of the wild type of Col-0 were sown on MS medium containing 100 mM NaCl and on MS control medium. In presence of 100 mM NaCl after 14d all genotypes showed reduced growth, when compared with plants grown on control medium (**Figure 1A**). Compared to Col-0, the *elf7* and *elf8* mutants were affected to a greater extent, while the *cdc73* mutants grew rather better than Col-0. These observations were reassessed with plants grown on control medium for seven days, before transfer onto control medium or onto medium containing 100 mM NaCl. The growth of the transferred plants confirmed the previous finding that *elf7-2/3* and *elf8-1/4* plants were more strongly affected by NaCl than Col-0, while *cdc73-1/2* were affected less than Col-0 (**Figure 1B**). The aerial parts of the different genotypes were quantified after growth in presence or absence of 100 mM NaCl. Only minor differences between the genotypes were observed on control medium regarding rosette diameter and fresh weight (**Figures 1C, D**). However, when grown in presence of 100 mM NaCl, rosette diameter and fresh weight of *elf7* and *elf8* plants were significantly reduced, while *cdc73* were somewhat bigger than Col-0. Furthermore, the relative growth of the primary root of the different genotypes was monitored on vertically oriented plates, comparing root elongation in presence of 100 mM NaCl with that of the same genotype on control medium (**Supplementary Figure S1**). Quantification of root length revealed that the roots of Col-0 and *cdc73* grew comparably, whereas the relative root growth of *elf7* and *elf8* plants was clearly decreased (**Figure 1E**). Thus, compared to the Col-0 wild type, exposure to 100 mM NaCl has a profound negative effect on the growth of *elf7* and *elf8*, while the lack of CDC73 apparently has no adverse effect on the tolerance to NaCl.

**Figure 2:**
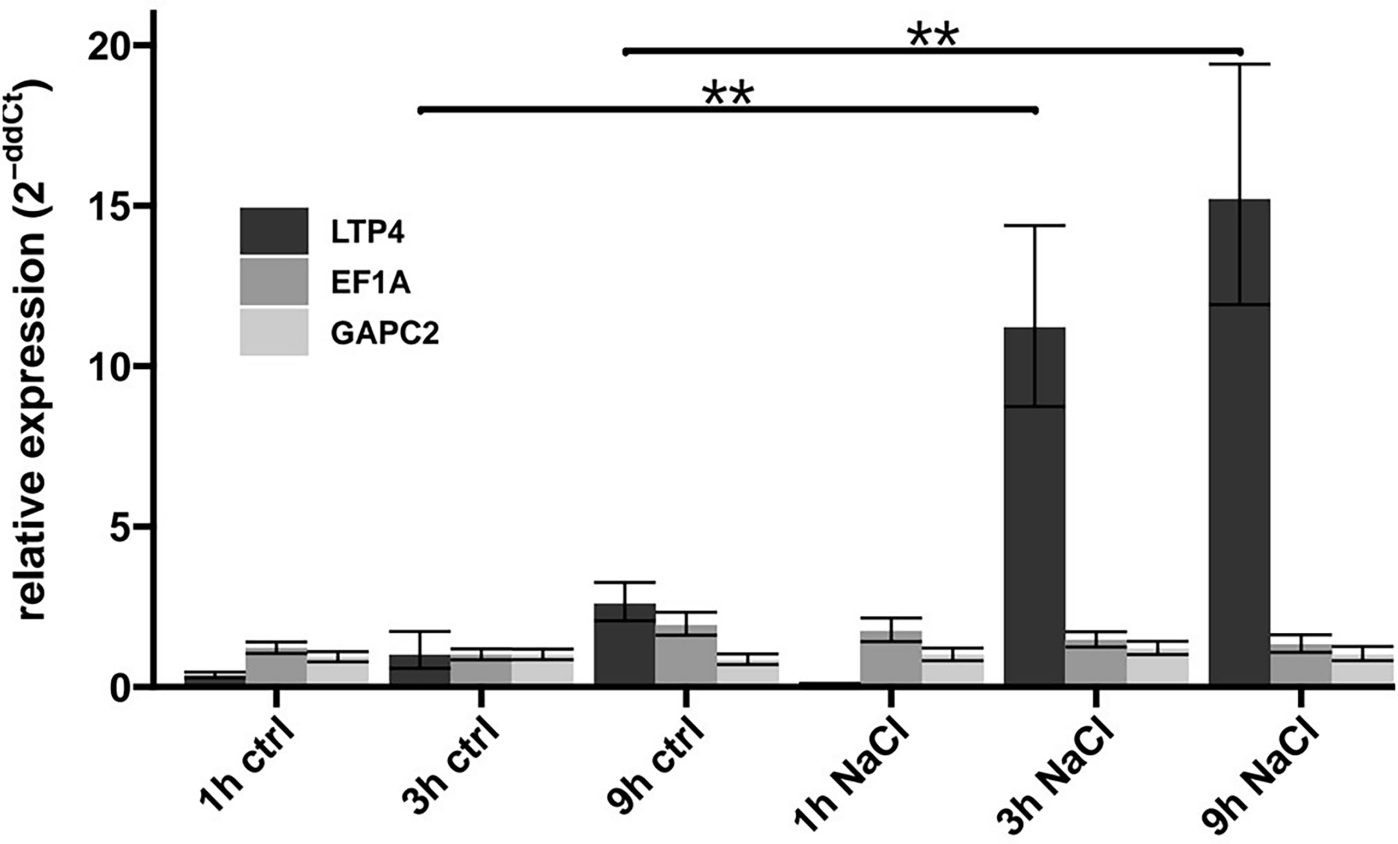
Changes in transcript levels upon exposure to NaCl. After growth on solid MS medium for 7 DAS, Col-0 plants were transferred to control medium (ctrl) or to medium containing 100 mM NaCl (NaCl). After different periods of the indicated treatment, RNA was isolated and the transcript levels of *LTP4* and reference genes (*EF1A*, *GAPC2*, *ACT2*) quantified by RT-qPCR. The depicted relative expression was normalised to the level of *ACT2* transcript in Col-0. The bars represent the mean relative expression of three technical replicates and error bars represent SD. The relative expression of *LTP4* was compared between control and NaCl treated condition of the same timepoints using Wilcoxon´s signed-rank test indicated by brackets (**, *p* ≤ 0.01).

To figure out the time it takes in our experimental setup until transcriptional induction is traceable, we determined the levels of the *LTP4* mRNA that is strongly up-regulated in *Arabidopsis* plants in response to NaCl (Winter et al., 2007). Using RT-qPCR, the *LTP4* transcript was quantified (relative to transcripts of three housekeeping genes) at different times after transfer of Col-0 plants onto medium containing 100 mM NaCl or control medium. Consistent with the induction kinetics of other studies (Kilian et al., 2007), the *LTP4* transcript, but not the two controls, were clearly up-regulated in our setup after 3h and 9h of exposure to 100 mM NaCl, while after 1h no induction was detected (**Figure 2**). For the following analyses addressing transcriptomic changes upon NaCl exposure, we focused on the three genotypes Col-0, *elf7-3* and *cdc73-2*.

### ELF7 is required for efficient transcriptional response upon NaCl exposure

To learn whether the differential response to NaCl exposure of *elf7-3* relative to Col-0 and *cdc73-2* is paralleled by distinct transcriptional changes, we performed genome-wide transcript profiling of these three genotypes using RNA-seq. We intended to generate information on transcriptional output (freshly synthesized unspliced/spliced mRNAs including nascent transcripts) rather than steady-state mRNA levels obtained with total poly(A) mRNA (Zaghlool et al., 2013; Dhaliwal and Mitchell, 2016). Therefore, we (i) isolated nuclear RNA (rather than total RNA) (**Supplementary Figure S2**), (ii) used rRNA depletion (rather than poly(A) enrichment) and (iii) made use of low RNA size cut-off (≥25 nt). Four biological replicates of RNA of each genotype with or without 3h exposure to 100 mM NaCl were used for preparation of sequencing libraries. Analysis of the RNA-seq data revealed >35 million unique reads per genotype (**Table S2**) and that the biological replicates of the analyzed genotypes/conditions yielded robust results (**Supplementary Figure S3**), except for one of the *elf7-3*/NaCl samples that was removed as outlier. Analysis of genes differentially expressed in *elf7-3* or *cdc73-2* compared to Col-0 under control conditions identified 12 and 722 differentially expressed genes (DEGs) in *cdc73-2* and *elf7-3*, respectively (**Supplementary Figure S4**). Gene ontology (GO) term analysis of the DEGs in *elf7-3* identified genes responsive to salt stress as a prominent term (**Supplementary Figure S5**), while due to the small number of DEGs in *cdc73-2* the analysis showed no significant enrichment. The differential expression of only subsets of genes is in general agreement with *Arabidopsis* mutants deficient in other TEFs (van Lijsebettens and Grasser, 2014), although the transcriptomic difference to wild type is remarkably low in case of *cdc73-2*.

**Figure 3:**
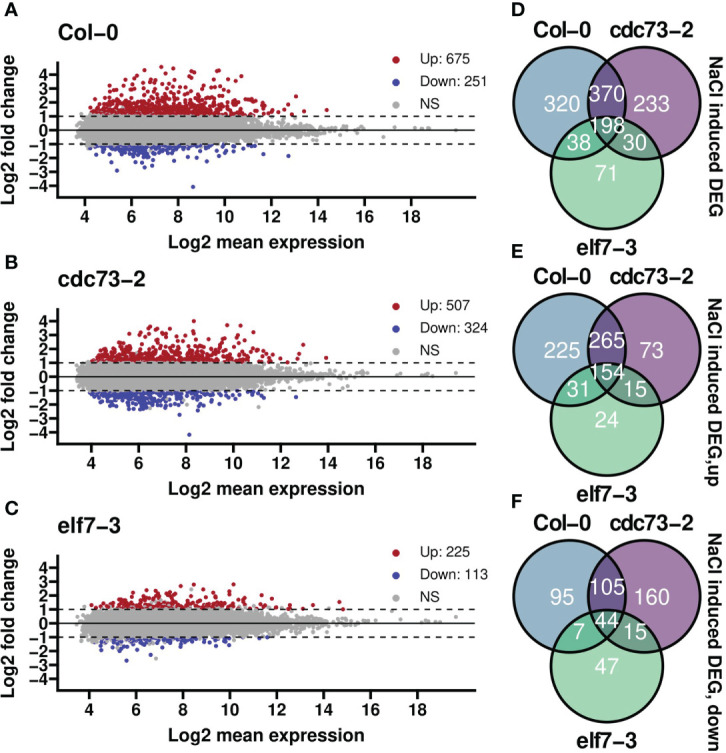
Differential gene expression analysis of *elf7-3* and *cdc73-2* relative to Col-0 upon NaCl treatment. **(A–C)** differential gene expression between treatment and control groups for each genotype separately. Highlighted in red are genes that are significantly upregulated after salt stress (log-fold change, LFC≥ 1 and *padjusted* ≤ 0.05), while in blue are genes that are significantly downregulated upon NaCl exposure (LFC≤ −1 and *padjusted* ≤ 0.05). NS = not significant. Numbers in the legends represent the gene counts in that group. **(D, E)** Venn diagrams summarising the number of differentially expressed genes in the different genotypes (as in **A–C**) upon NaCl treatment with total number of differentially expressed genes **(D)**, upregulated **(E)** or downregulated **(F)** genes. ns, not significant.

Analysis of differential gene expression with or without NaCl treatment for the genotypes separately revealed numerous significantly up- or down-regulated genes in Col-0 and *cdc73-2* (**Figures 3A, B**). In contrast, a clearly lower number of DEGs with mostly smaller expression changes was detected in *elf7-3* (**Figure 3C**). Principal component analysis (PCA) revealed a strict clustering of the samples according to NaCl treatment and control treatment (**Supplementary Figure S6**) that together with the observed Pearson correlation (**Supplementary Figure S3**) indicates the reproducibility of the obtained data. The transcriptomic analysis illustrates that 926 and 831 nuclear-encoded genes are differentially expressed upon NaCl treatment in Col-0 and *cdc73-2*, respectively (**Figure 3D**). A major part (568 genes) of the DEGs appears to be equally regulated in both genotypes. Moreover, a greater number of genes is up-regulated in both genotypes (675 and 507 for Col-0 and *cdc73-2*, respectively) when compared to the down-regulated genes (251 and 324 for Col-0 and *cdc73-2*, respectively) (**Figures 3D, E**). In *elf7-3*, 338 DEGs are detected upon NaCl treatment, of which 225 and 113 are up- and downregulated, respectively.

**Figure 4:**
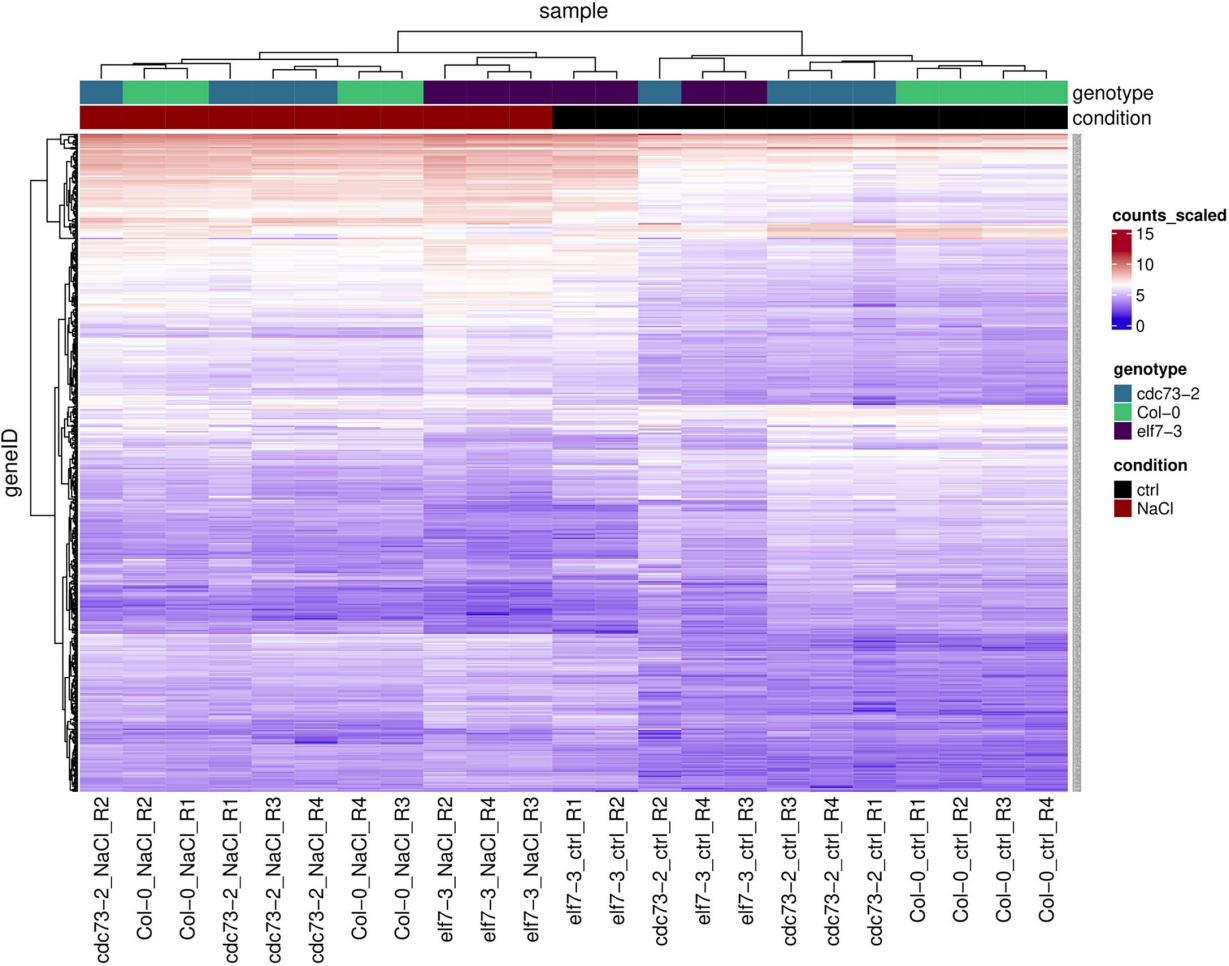
Heatmap and hierarchical clustering of the 500 most variable genes identified by transcript profiling of *elf7-3* and *cdc73-2* relative to Col-0 upon NaCl treatment. These genes were selected from the scaled counts of the count table using the subread package “featureCounts” and the count table includes the counts for all replicates, conditions and genotypes. Relative transcript abundance is shown as heatmap using the indicated scale (LFC of scaled counts). Genotypes and condition (ctrl: control; NaCl: 3h 100 mM NaCl) are colour-coded on top.

Hierarchical clustering analysis of the 500 genes that showed most variable transcript levels upon exposure to NaCl was visualized as a heatmap. Here, in agreement with the PCA (**Supplementary Figure S6**), predominantly two clusters are apparent, representing the two different conditions “control” and “NaCl” (**Figure 4**). As shown above, compared to Col-0 and *cdc73-2*, *elf7-3* exhibit decreased changes in transcript levels upon exposure to NaCl. Further analysis revealed that both the potential of *elf7-3* plants to induce and to repress transcription is decreased (**Supplementary Figure S7**). The NaCl-induced transcriptomic changes, occurring in Col-0 upon exposure to NaCl (926 DEGs) were further examined by GO term analysis. As expected (Golldack et al., 2014; Yang and Guo, 2018), this analysis demonstrated predominant enrichment of the GO term “response to salt stress” in all three genotypes (**Supplementary Figure S8**).

**Figure 5:**
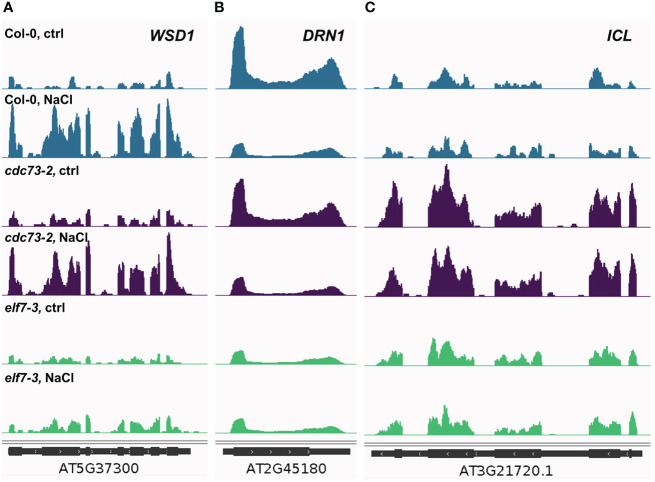
Changes in transcript levels in the different genotypes at individual loci dependent on the presence of NaCl. Coverage of merged RNA-seq samples was normalised to the effective *Arabidopsis* genome size and visualised using the Integrative Genomics Viewer. **(A)** coverage of *WSD1* (At5g37300), a gene induced by elevated salt concentrations. **(B)** coverage of *DRN1* (At2g45180), a gene repressed by elevated salt concentrations. **(C)** coverage of *ICL* (At3g21720), a gene, whose expression enhances salt tolerance.

The differential transcriptional response observed with the different genotypes is also evident at the level of individual genes. As exemplified by the NaCl-inducible wax synthase/acyl-CoA:diacylglycerol acyltransferase (*WSD1*) gene, which plays a critical role in wax ester synthesis (Abdullah et al., 2021). Upon NaCl exposure substantially increased transcript levels of *WSD1* are detected in Col-0 and *cdc73-2*, while expression of the gene is hardly altered in *elf7-3* (**Figure 5A**). Another situation is observed for NaCl-repressed *DISEASE RELATED NONSPECIFIC LIPID* 26 *TRANSFER PROTEIN 1* (*DRN1*) gene, required for defense against pathogens as well as for normal seedling growth under salinity stress (Dhar et al., 2020). The *DRN1* transcript levels are reduced in presence of NaCl in Col-0 and *cdc73-2*, but not in *elf7-3* (**Figure 5B**). In case of the *ICL* gene encoding isocitrate lyase that plays a role in plant salt tolerance through the glyoxylate cycle (Yuenyong et al., 2019), no transcriptional change occurs in response to NaCl. However, relative to Col-0 and *elf7-3* distinctly elevated *ICL* transcript levels are detected in *cdc73-2* (**Figure 5C**). As discussed below, the differential transcriptional response might provide an explanation for the major difference of the three genotypes regarding their tolerance towards NaCl.

**Figure 6:**
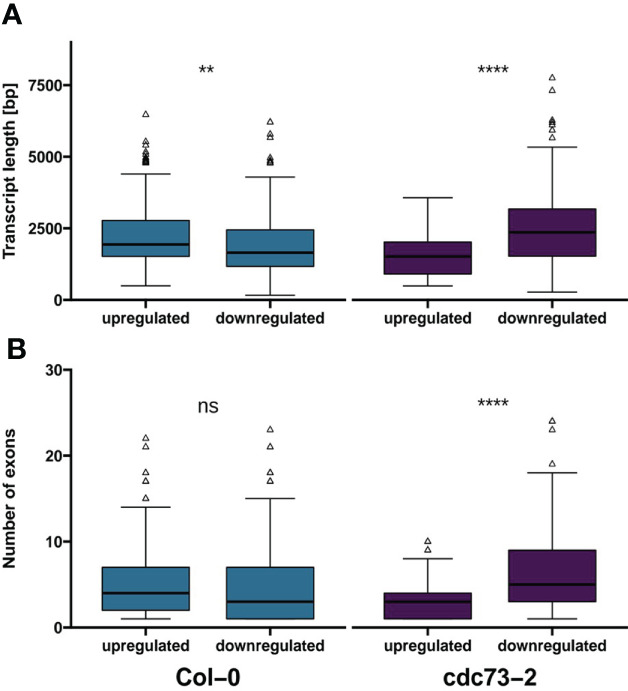
Transcript length and number of exons of genes uniquely differentially expressed in Col-0 or *cdc73-2*. **(A)** average transcript length of genes differentially expressed either in Col-0 or *cdc73-2*. **(B)** number of exons of genes differentially expressed either in Col-0 or *cdc73-2*. Data was evaluated using Wilcoxon signed-rank test (**, *p* ≤ 0.01; ****, *p* ≤ 0.0001; ns, not significant).

Further evaluation of genes specifically up- or down-regulated upon NaCl treatment in Col-0 or *cdc73-2* revealed that in Col-0 the average length of up-regulated transcripts is greater than that of the down-regulated transcripts, whereas the inverse is seen in *cdc73-2* (**Figure 6A**). The number of exons in these genes is higher in genes down-regulated in *cdc73-2* relative to the up-regulated genes, while regarding number of exons there is no significant difference among the DEGs specifically regulated in Col-0 (**Figure 6B**). Together these findings suggest that in the absence of CDC73 the processivity of RNAPII is decreased, which is particularly relevant for the transcription of longer genes. Analysis of differential splicing events induced upon NaCl exposure in either *cdc73-2* or Col-0 revealed only very few events (**Supplementary Figure 9**). This suggests that the greater length of down-regulated genes does not correlate with differential splicing events, but rather with a reduced transcriptional processivity, albeit the sequencing depth and read length of the experimental setup was not sufficient for a comprehensive spicing analysis.

**Figure 7:**
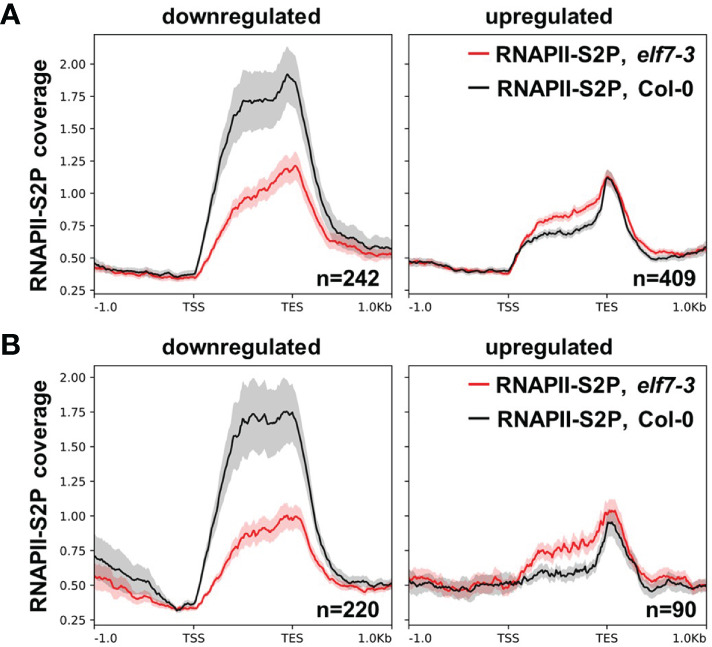
RNAPII coverage over genes up- and downregulated in *elf7-3*. Metagene plots of ChIP-seq analysis of Col-0 and *elf7-3* using an antibody directed against RNAPII-S2P over DEGs elf7-3/Col-0 under standard condtions **(A)** or upon exposure to NaCl **(B)**. Mean signals of the three biological replicates were averaged (line) and the shaded area represents the SEM for the three replicates at each position. The tracks were scaled over transcribed regions from transcriptional start site (TSS) to transcriptional end site (TES).

Chromatin immunoprecipitation (ChIP) in combination with high throughput sequencing (ChIP-seq) was employed to examine the distribution of RNAPII over genes, whose expression is dependent on the presence/absence of ELF7. Chromatin of Col-0 and *elf7-3* was analyzed with an antibody specific for elongating RNAPII (RNAPII-S2P). Analyses of the ChIP-seq data revealed 12.3-18.8 million high quality reads per genotype and the biological replicates show robust correlation (**Supplementary Figure S10**). RNAPII coverage was compared for genes up- or downregulated in *elf7-3 vs*. Col-0 under standard conditions or upon exposure to NaCl. For upregulated genes RNAPII coverage is mildly increased, whereas for downregulated genes RNAPII coverage is clearly reduced in *elf7-3* (**Figure 7**). The genes with highly reduced RNAPII coverage include, for instance, also the above-mentioned *DRN1* gene (cf. **Figure 5**), whose expression is required for salt tolerance (Dhar et al., 2020). Therefore, ELF7 is particularly necessary for transcription of genes mis-regulated in *elf7-3* and transcriptional upregulation appears to depend more strongly on ELF7 (cf. **Supplementary Figure S4**).

## Discussion

PAF1C travels with RNAPII to regulate transcript elongation and to modulate chromatin structure of transcribed regions (Jaehning, 2010; Francette et al., 2021). In plants, so far mainly the role of PAF1C in developmental processes was studied (He et al., 2004; Oh et al., 2004; Park et al., 2010; Yu and Michaels, 2010; Fal et al., 2017). In few cases the observed mutant phenotype(s) could be correlated with misexpressed target genes, such as the above-mentioned link between *FLC* expression and time of flowering (Zhang et al., 2003; He et al., 2004; Oh et al., 2004; Xu et al., 2008; Park et al., 2010). Generally, *Arabidopsis* mutants deficient in various TEFs (e.g. Elongator, SPT4-SPT5, TFIIS) exhibit differential expression of only subsets of genes (Nelissen et al., 2005; Grasser et al., 2009; Dürr et al., 2014). Likewise, relative to wild type under normal growth conditions, based on microarray hybridization experiments (Park et al., 2010) or RNA-seq of total mRNA (Nasim et al., 2022), several hundreds of DEGs were observed for mutants deficient in PAF1C subunits, except for *cdc73*, whose transcriptome was similar to that of wildtype. Consistently, using high-throughput sequencing of nuclear mRNAs, we identified 12 and 722 DEGs in *cdc73-2* and *elf7-3*, respectively. Although it is difficult to directly compare transcriptomics datasets obtained with different techniques and analyzed using distinct statistical methods, the results in general support the emerging view that in plants TEFs are required for the correct transcription of only subsets of genes (van Lijsebettens and Grasser, 2014).

Recently, PAF1C (i.e. VIP3, VIP5, VIP6) was also identified as a critical factor in the plant response to repeated mechanic stimulation (Jensen et al., 2017) and in temperature-responsive flowering (Nasim et al., 2022). We have comparatively exposed different PAF1C subunit mutants to elevated concentrations of NaCl to provoke transcriptional response. In presence of NaCl, *elf7* and *elf8* displayed more severe decrease in growth of the aerial parts than Col-0, whereas the rosette size of *cdc73* rather exceeded that of Col-0. Growth of the primary root upon NaCl exposure was similarly decreased in Col-0 and *cdc73-1/cdc73-2*, whereas root growth of *elf7-*2/*elf7-3* and *elf8-1/elf8-4* was considerably more strongly reduced. Recently, it was reported – based on a similar, albeit not identical experimental setup – that the root growth of *elf7-3* in presence of 100 mM NaCl was comparable to that of Col-0 (Li et al., 2019). In view of that we accurately measured the relative root growth of both *elf7* alleles in presence of NaCl relative to their growth on control medium, taking into consideration the distinct growth of the mutants compared to Col-0 in absence of NaCl. Thereby, compared to the other genotypes we determined significantly decreased root growth of *elf7-2* and *elf7-3* (and *elf8-1/elf8-4*) in presence of NaCl.

To record rather early transcriptional responses, we profiled the transcriptomes of *elf7-3* and *cdc73-2* in comparison to Col-0 after 3h of NaCl exposure, analyzing nuclear mRNAs including nascent transcripts. Col-0 and to a slightly lesser extent *cdc73-2* exhibited clear transcriptomic changes upon NaCl exposure, whereas *elf7-3* showed a comparatively clearly decreased response to this stress condition (**Figure 3**) that is also reflected by an altered RNAPII coverage in *elf7-3* (**Figure 7**). A major part of the genes differentially expressed in Col-0 upon NaCl exposure were also differentially expressed in *cdc73-2*, illustrating that both genotypes respond similarly to the presence of NaCl. With *elf7-3* plants, the presence of NaCl resulted in distinctly lower transcriptional response, indicating that ELF7 plays an important role in the response to elevated NaCl concentrations. In agreement with our findings, a recent study concluded that Col-0 and *cdc73 Arabidopsis* plants grown at 23°C have a similar transcriptome, and that *elf7* and *vip3/4/5/6* share a large number of DEGs (Nasim et al., 2022). In yeast as well as in mammals, PAF1C subunit mutants are also associated with a range of phenotypes suggesting (partial) subunit specificity (Betz et al., 2002; Akanuma et al., 2007; Chu et al., 2007; Cheung et al., 2008; Wang et al., 2008). Furthermore, yeast PAF1C subunit mutants exhibit various defects in the tolerance to environmental stress conditions (Shi et al., 1996; Betz et al., 2002; García et al., 2016). Particularly, inactivation of PAF1 and CTR9 result in severe mutant phenotypes in accord with their central structural role within PAF1C (Kim et al., 2010; Chu et al., 2013; Vos et al., 2020). Moreover, the different PAF1C subunits are involved to a variable extent in modulating the genomic distribution of a range of histone marks including H2B mono-ubiquitination and various transcription-related H3 methylations (Jaehning, 2010; Francette et al., 2021).

Analysis of the genes that are differentially expressed upon exposure of *Arabidopsis* plants to NaCl demonstrated that a greater part of the DEGs is comparably regulated in Col-0 and *cdc73-2*, illustrating that a similar transcriptional response occurs in both genotypes. In contrast, a comparatively lower transcriptional response can be observed with *elf7-3*, suggesting that ELF7 is crucial for this type of stress response, and accordingly *elf7-3* (and *elf7-2*) plants are rather susceptible to NaCl. At the same time the above-mentioned finding that the expression of salt-responsive genes is altered in *elf7-3* plants under control conditions, may influence the adaptability of these plants upon exposure to NaCl, although the mechanism, how the lack of ELF7 influences gene expression under these conditions remains unknown. Interestingly, the tolerance to NaCl of *cdc73-2* plants is even more pronounced than that of Col-0. Elevated expression of gene(s) that enhance the tolerance to NaCl such as the *ICL* gene (Yuenyong et al., 2019) could contribute to the improved performance of *cdc73* plants under conditions of increased salt concentrations. The *ICL* transcript is not regulated by the presence of NaCl, but there are significantly elevated levels of the transcript in *cdc73-2* relative to Col-0 and *elf7-3* (**Figure 5C**) that may augment the resistance of *cdc73-2* to NaCl. However, several non-overlapping DEGs in *cdc73-2* represent salt-responsive genes (**Supplementary Figure S11**) that may also contribute to the salt tolerance of *cdc73-2*. Examination of DEGs that are specifically regulated in Col-0 or *cdc73-2* revealed a greater average length of the transcripts down-regulated in *cdc73-2* compared to Col-0. In line with that the genes down-regulated in *cdc73-2* contain a greater number of exons. Our comparative analysis of alternative splicing events in Col-0 and *cdc73-2* revealed only few events, but we consider this outcome rather inconclusive. Together these findings suggest that in the absence of CDC73 the processivity of RNAPII is decreased, which is particularly relevant for the transcription of longer genes. In line with that, depletion of PAF1C in mouse cells results in decreased RNAPII processivity and reduced elongation rate (Hou et al., 2019). In conclusion, our study demonstrates that the *Arabidopsis* PAF1C subunit mutants deficient in ELF7 and CDC73 respond very distinctly to NaCl, which is reflected by the different transcriptional response. Plants lacking CDC73 transcriptionally respond to NaCl similar to Col-0, but still – likely because of altered expression of certain gene(s) – exhibit increased tolerance to NaCl. In contrast, the transcriptional response of plants lacking ELF7 is decreased and consequently the plants are markedly sensitive to NaCl exposure. Therefore, our analyses provide evidence for PAF1C subunit specificity in plant response to environmental conditions.

## Data availability statement

The data presented in the study are deposited in the Sequence Read Archive (SRA) repository, accession number PRJNA816434 (https://www.ncbi.nlm.nih.gov/bioproject/PRJNA8164).

## Author contributions

SO, RS, and TS performed the experimental procedures; SO, RS, US, and CM analyzed the next-generation sequencing data; SO, CM, and KDG designed the research; KDG wrote the manuscript and all authors approved the submitted version. All authors contributed to the article and approved the submitted version.

## Funding

This research was supported by the German Research Foundation (DFG) through grants Gr1159/14-2 and SFB960/A6 to KDG.

## Acknowledgments

We thank Mathias Gradl for contributions to the project and the Nottingham Arabidopsis Stock Centre (NASC) for providing *Arabidopsis* T-DNA insertion lines.

## Conflict of interest

The authors declare that the research was conducted in the absence of any commercial or financial relationships that could be construed as a potential conflict of interest.

The reviewer IL declared a past co-authorship with one of the authors KDG to the handling editor.

## Publisher’s note

All claims expressed in this article are solely those of the authors and do not necessarily represent those of their affiliated organizations, or those of the publisher, the editors and the reviewers. Any product that may be evaluated in this article, or claim that may be made by its manufacturer, is not guaranteed or endorsed by the publisher.
