# Supplementary material for: Distinct role of subunits of the Arabidopsis RNA polymerase II elongation factor PAF1C in transcriptional reprogramming
Source: Front Plant Sci. 2022 Sep 29;13:974625. doi: 10.3389/fpls.2022.974625 (PMC9558118; doi:10.3389/fpls.2022.974625)
Supplement: Supplementary file 1 [file DataSheet_1.pdf]

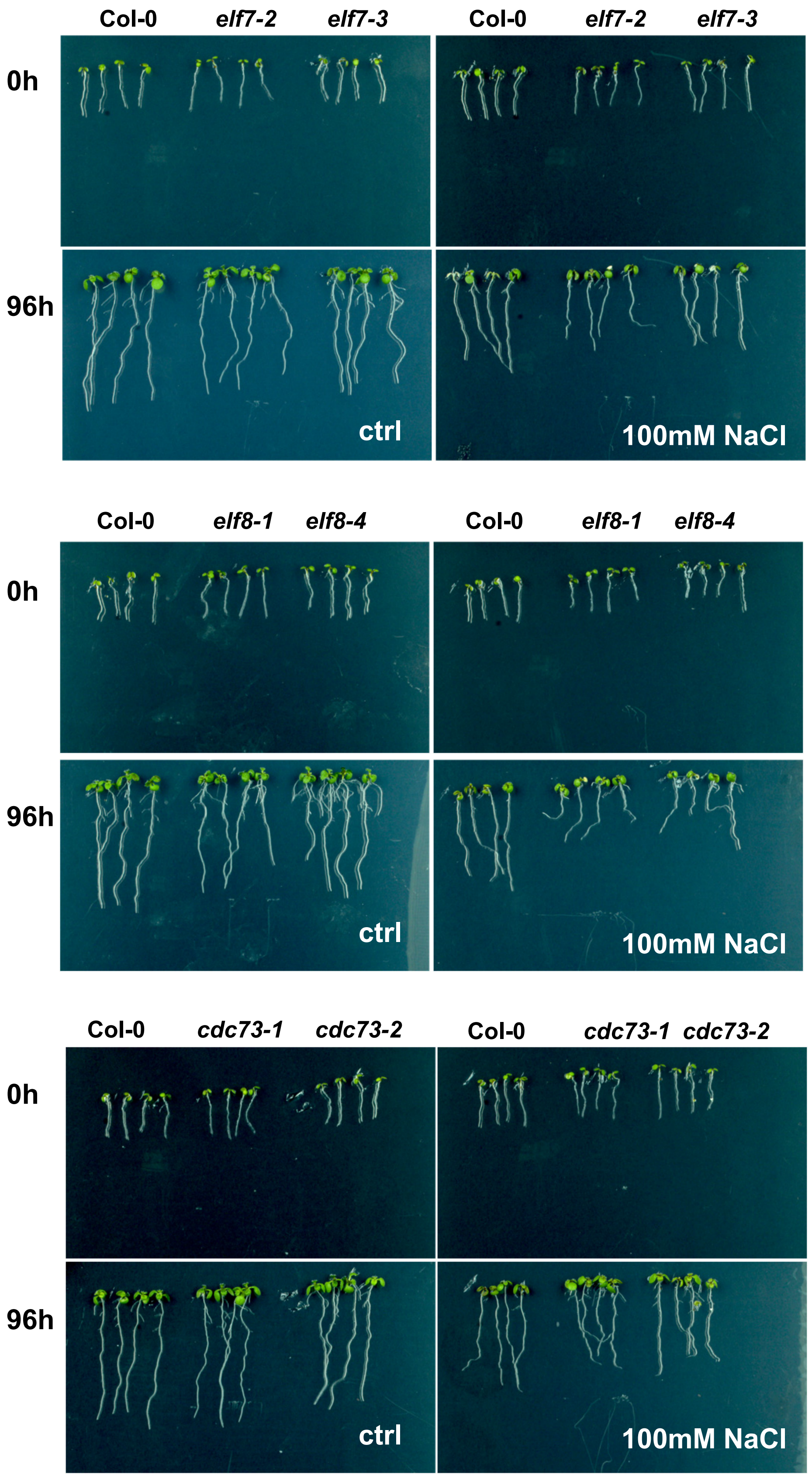

Fig. S1. Root growth of the different genotypes in absence (ctrl) or presence of 100 mM NaCl. Plants of the indicated genotypes were grown on vertical MS plates for 7 DAS, before they were transferred to control medium or medium containing 100 mM NaCl. Representative plants were documented 0h or 96h after transfer.

A

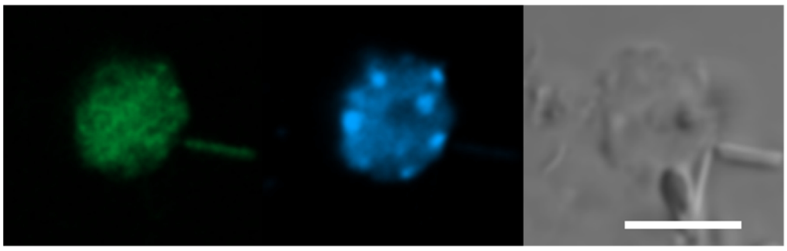

B

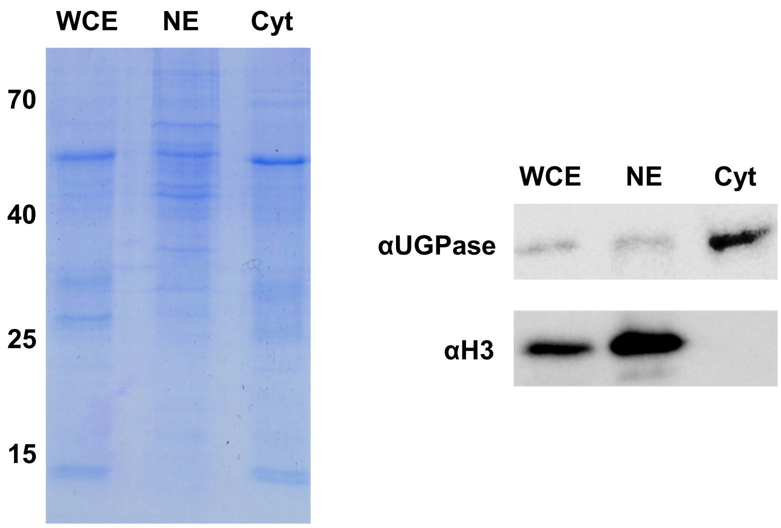

Fig. S2. Microscopic and immunoblot analysis of isolated nuclei. **A**, to assess the suitability of the nuclei isolated from 7-DAS plants as source for nuclear RNAs, nuclei were analysed by confocal laser scanning microscopy (CLSM). For test purposes nuclei were isolated from transgenic plants expressing eGFP-NLS (eGFP fused to a nuclear localisation signal, Pfab et al., 2018) and analysed by CLSM. Nuclei were visualised by means of GFP fluorescence (left) and 4′,6-diamidino-2-phenylindole (DAPI) fluorescence (middle). The corresponding brightfield image is shown (right) along with a size bar representing 5 µm. **B**, whole cell extract (WCE), nuclear pellet (NE) and supernatant of the centrifugation, essentially corresponding to cytoplasm (Cyt) were separated by SDS-PAGE and stained with Coomassie (left) or analysed by immunoblotting with antibodies directed against nuclear histone H3 or cytoplasmic UGPase (right).

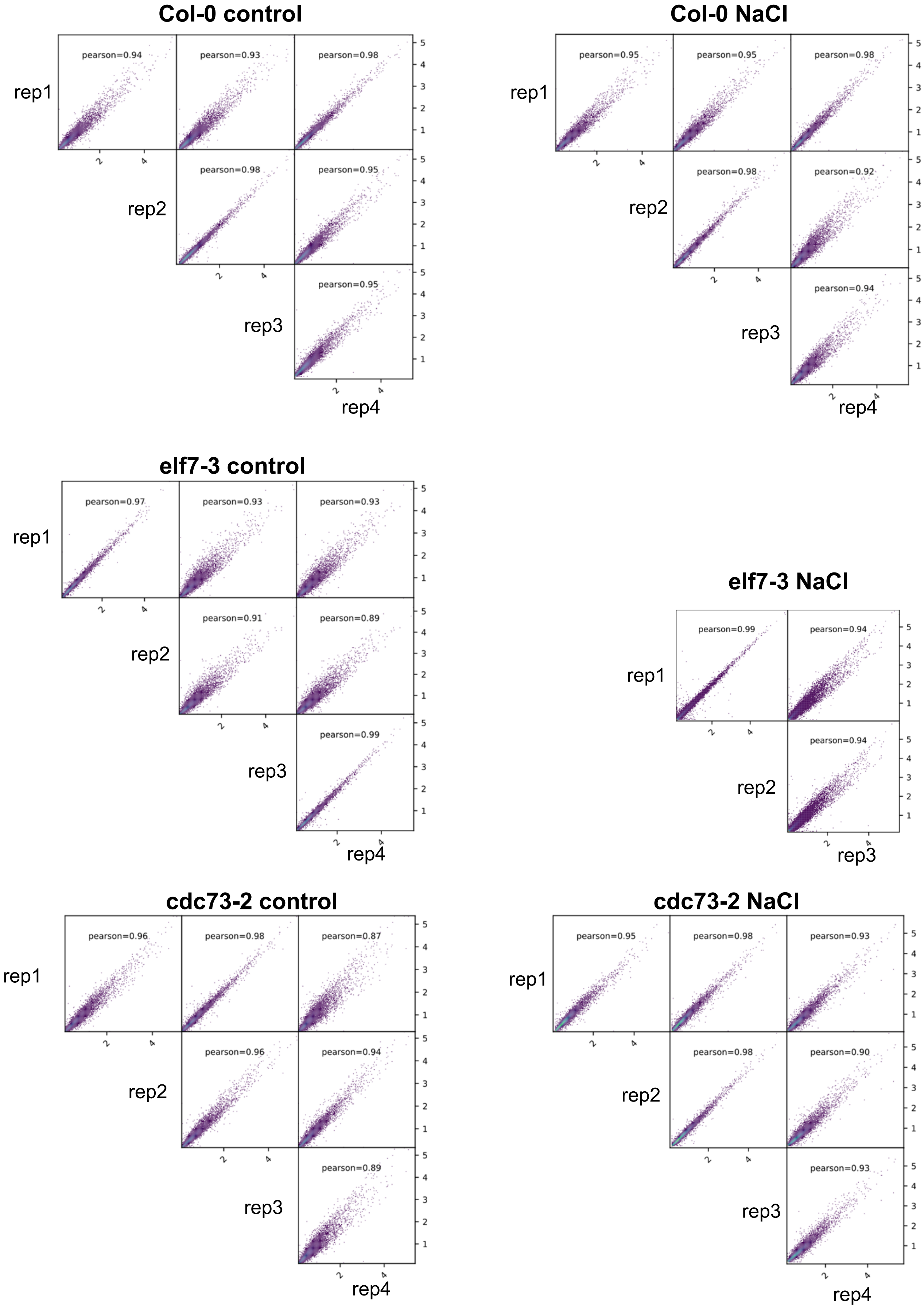

Fig. S3. RNA-Seq replicates show high pairwise correlation. Pearson correlation between all replicates was calculated over 100 bp bins.

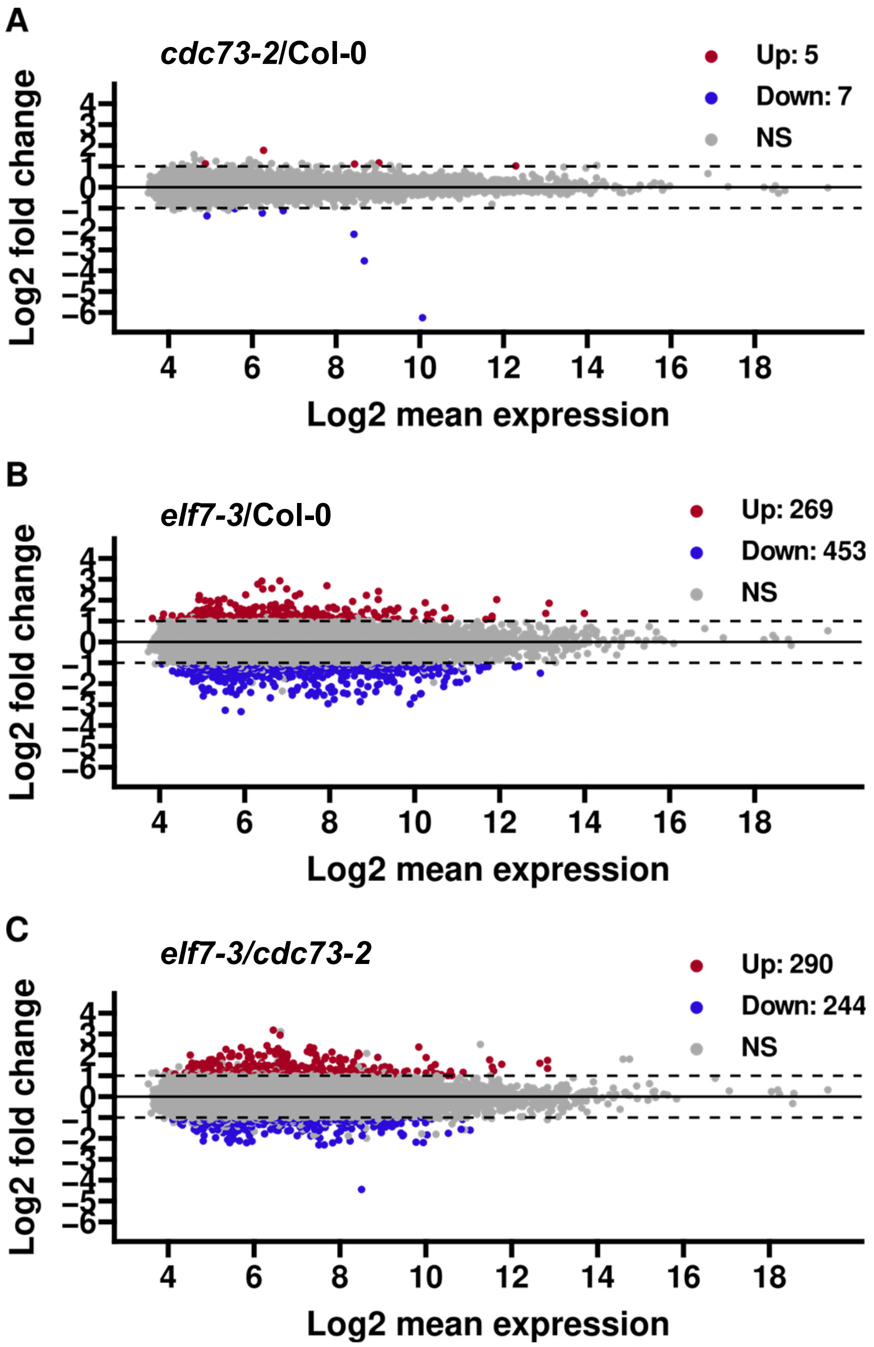

Fig. S4. Differential gene expression analysis of *elf7-3* and *cdc73-2* and Col-0 relative to each other under control conditions. **A-C**, differential gene expression between the indicated genotypes. Highlighted in red are genes that are significantly upregulated (log-fold change, LFC≥ 1 and *padjusted* ≤ 0.05), while in blue are genes that are significantly downregulated (LFC≤ −1 and *padjusted* ≤ 0.05). NS = not significant. Numbers in the legends represent the gene counts in that group.

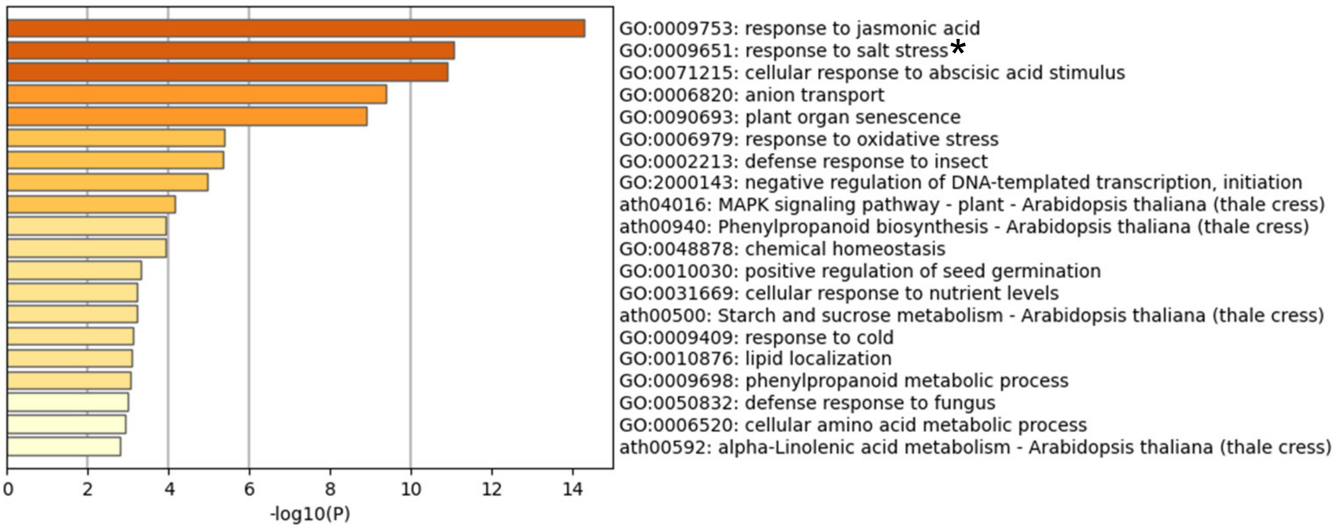

Fig. S5. Analysis of GO term enrichment using MetaScape of DEGs in *elf7-3* under standard conditions (n = 722).

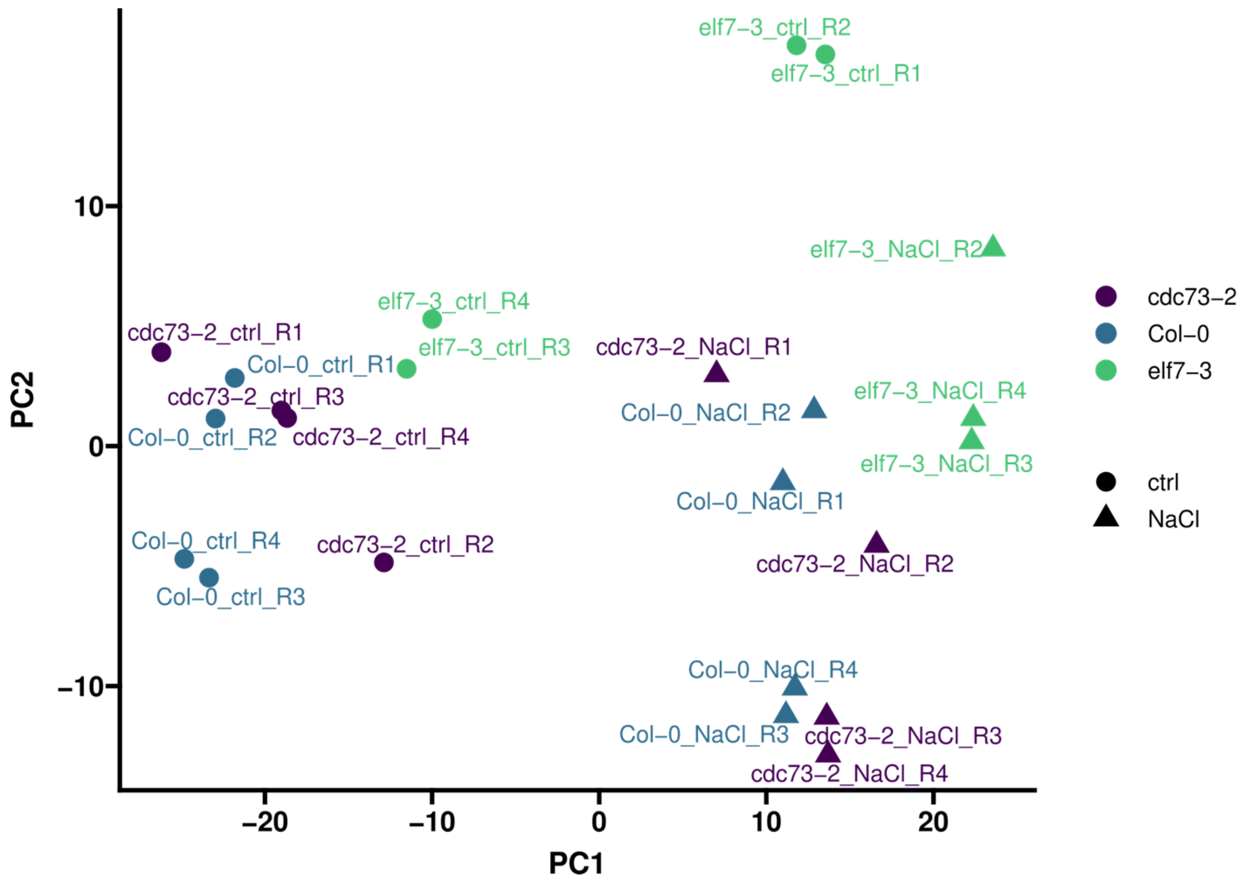

Fig. S6. Principal component analysis (PCA) of the RNA-seq data. Relative transcript abundance in each sample was summarised in a count table using the featureCounts function of the rsubread package. PCA was created using the reduce_dimensions function of the tidybulk R package on the abundance-scaled read counts using the 500 most variable genes. PC1 is the most variable principal component and PC2 is the second most variable component. The sample designation is provided in the legend.

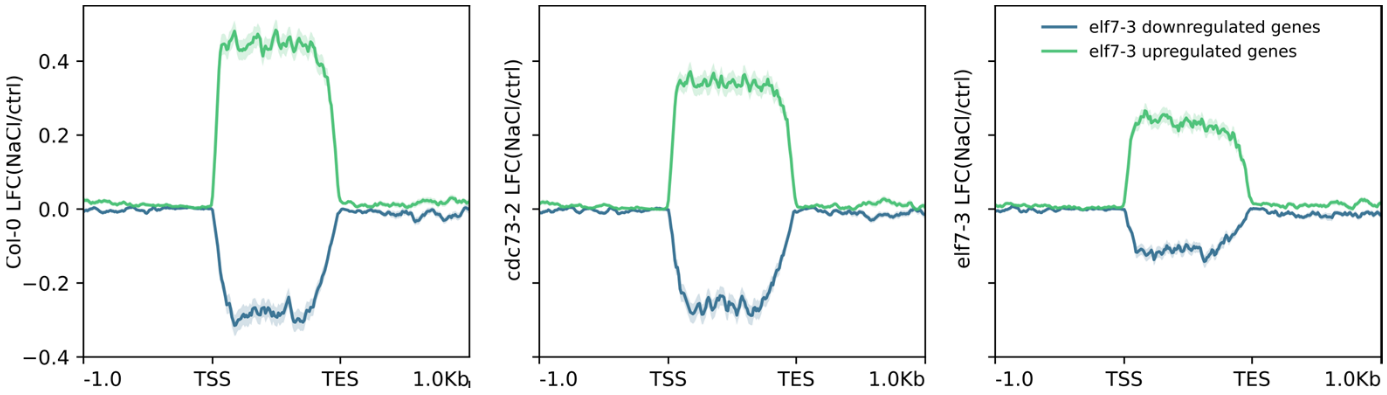

Fig. S7. Metagene plots of DEGs illustrate the distinct potential of the various genotypes to respond to the exposure to NaCl. Background/control-normalised RNA-seq tracks were plotted over DEGs up-/down-regulated in *elf7-3* vs. Col-0 under control conditions (n = 409 and n = 242, respectively). Differential coverage is detected over the entire transcribed region from transcriptional start sites (TSS) to transcriptional end site (TES). Upon exposure to NaCl *elf7-3* has a reduced capability to up- and downregulate genes.

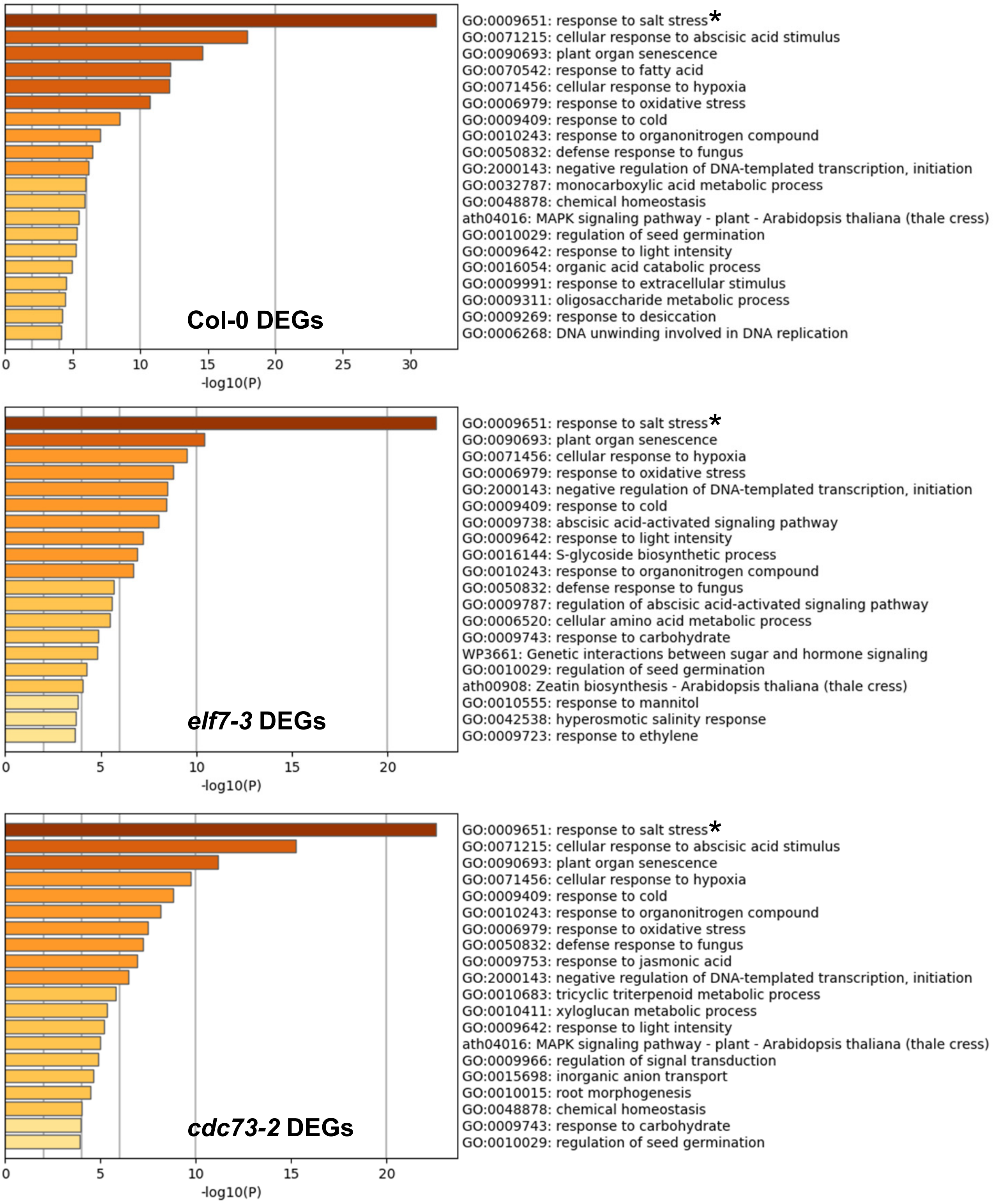

Fig. S8. Analysis of GO term enrichment using MetaScape of DEGs in Col-0, *elf7-3* and cdc73-2 upon exposure to 100 mM NaCl, demonstrating predominant enrichment of the term “response to salt stress”.

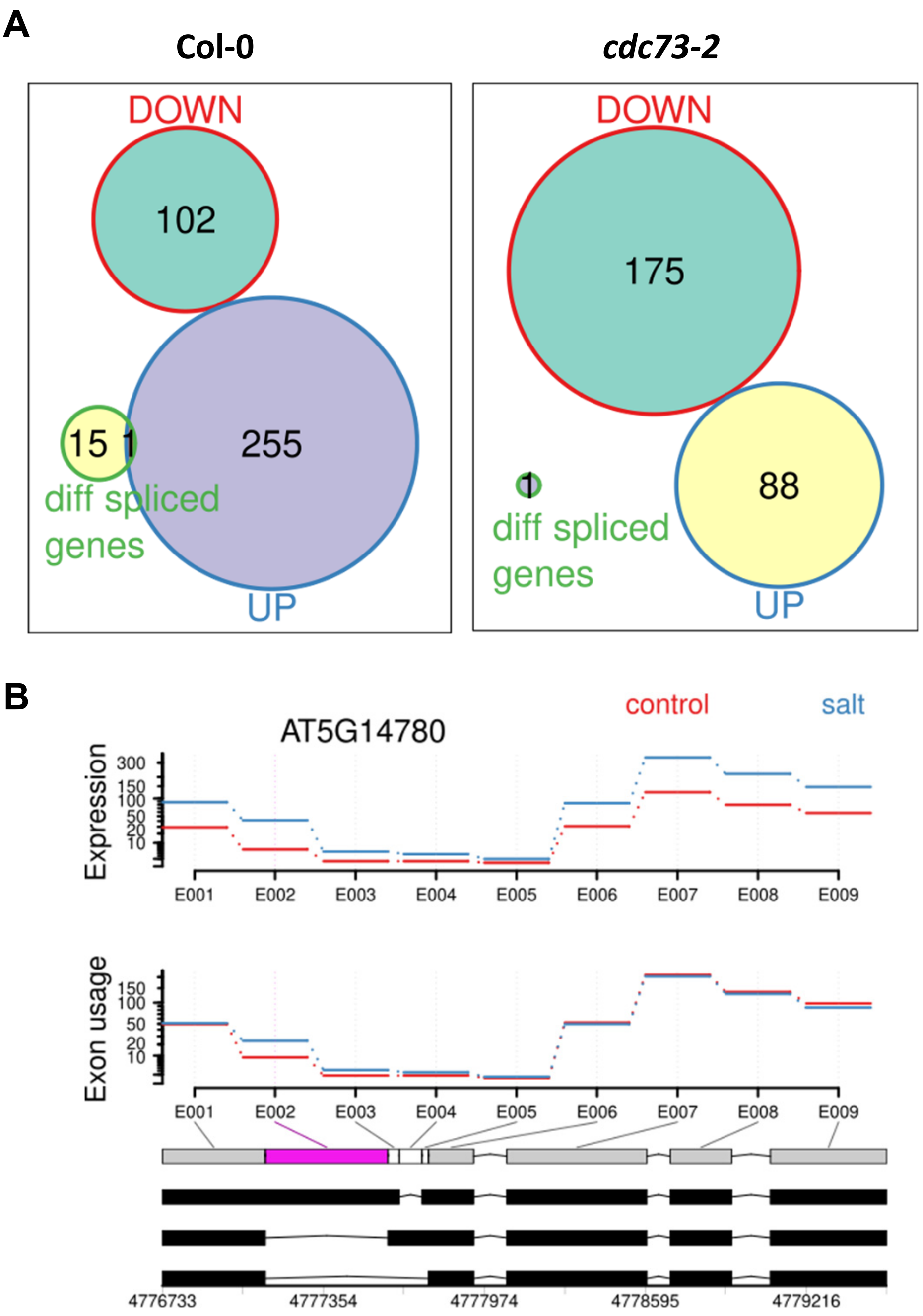

Fig. S9. Differential splicing analysis in *cdc73-2* and Col-0 upon NaCl exposure using DEXSeq. **A**, only very few significantly differentially spliced genes between salt and control conditions were detected (16 in Col-0; 1 in *cdc73-2*; FDR < 0.05). Furthermore those differential splice events do rarely/not overlap with uniquely up- or downregulated genes. **B**, the expression and exon usage of the one gene (AT5G14780), which is uniquely upregulated and differentially spliced in Col-0 is shown.

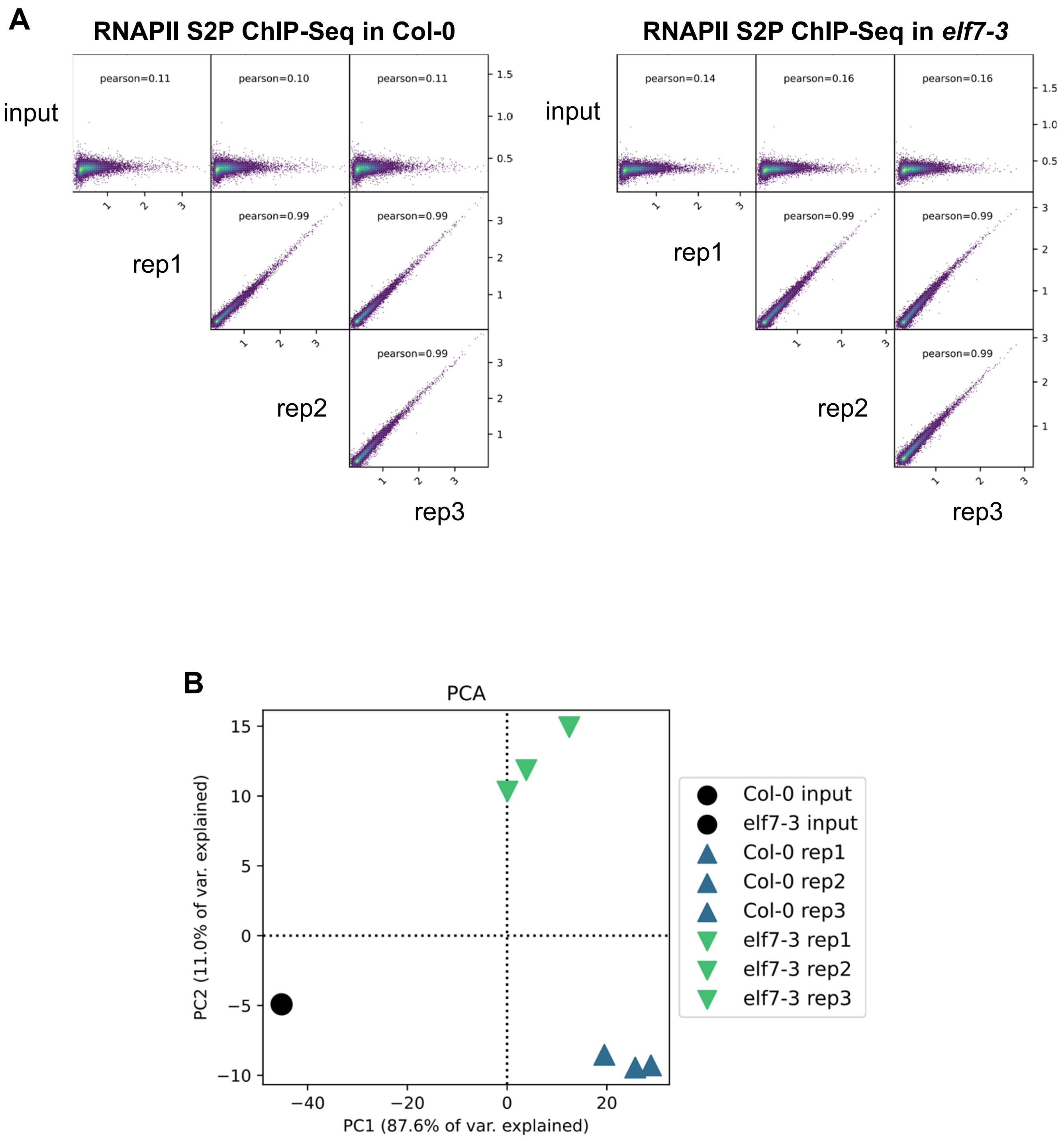

Fig. S10. **A**, ChIP-Seq replicates show high pairwise correlation. Pearson correlation between all replicates was calculated over Tair10 annotated non-overlapping protein coding genes. **B**, PCA of ChIP-Seq replicates over TAIR10 annotated non-overlapping protein coding genes. Replicates cluster according to genotype

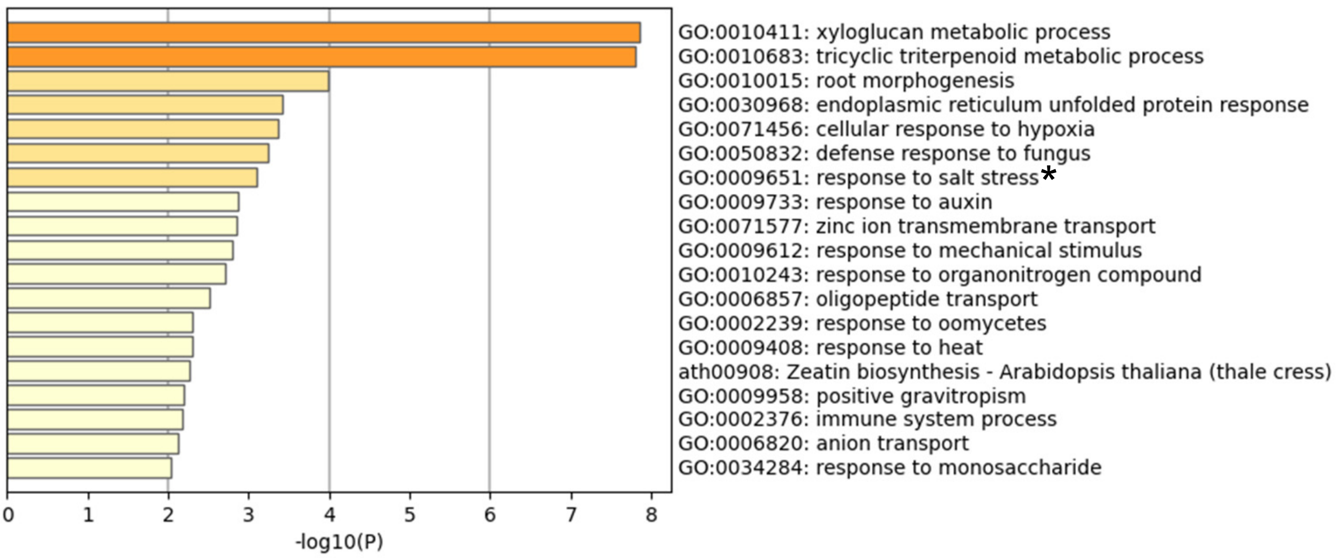

Unique *cdc73-2* DEGs in presence of 100 mM NaCl include salt-responsive genes:

AT5G37300	WSD1	O-acyltransferase (WSD1-like) family protein
AT1G09950	RAS1	RESPONSE TO ABA AND SALT 1
AT1G69430	AT1G69430	Son of sevenless protein
AT1G19380	AT1G19380	sugar, putative (DUF1195)
AT5G10730	AT5G10730	NAD(P)-binding Rossmann-fold superfamily protein
AT1G08930	ERD6	Major facilitator superfamily protein
AT1G77200	AT1G77200	Integrase-type DNA-binding superfamily protein
AT5G41740	AT5G41740	Disease resistance protein (TIR-NBS-LRR class) family
AT5G50720	HVA22E	HVA22 homologue E
AT1G70170	MMP	matrix metalloproteinase
AT5G24090	CHIA	chitinase A
AT5G43260	AT5G43260	chaperone protein dnaJ-like protein
AT2G42540	COR15A	cold-regulated 15a
AT5G46830	NIG1	calcium-binding transcription factor NIG1
AT1G50960	GA2OX7	gibberellin 2-oxidase 7
AT4G25670	AT4G25670	stress response NST1-like protein

Fig. S11. Analysis of GO term enrichment using MetaScape of non-overlapping DEGs in *cdc73-2* upon exposure to 100 mM NaCl conditions (n = 220) and list of salt-responsive DEGs.

Table S1. Oligonucleotides used in this study for genotyping and qRT-PCR

**Sequence**	**Usage**	**Target**
ATTTTGCCGATTTCGGAAC	Genotyping (ID 2368)	SALK T-DNA insertion
GCCTTCGTCCTCTAATCTTCTC	Genotyping (ID 1621)	T-DNA insertion *elf7-3* (SALK_019433)/AT1G79730
ACGAGCTCTGACCTCATACTC	Genotyping (ID 1620)	T-DNA insertion *elf7-2 (*SALK_046605*)*/AT1G79730
GCAAGCGTTTGATCGCGTT	Genotyping (ID 1622)	T-DNA insertion *elf8-1 (*SALK_090130)/AT2G06210
GGGGAATTCAGCAGAAGAGTG	Genotyping (ID 1623)	T-DNA insertion *elf8-1 (*SALK_090130*)*/AT2G06210
GTGTCCTTGTACCCGA	Genotyping (ID 5124)	T-DNA insertion *elf8-4 (*GK_270G12.01*)*/AT2G06210
CTTGCATGAATTCTTTGG	Genotyping (ID 5173)	T-DNA insertion *elf8-4 (*GK_270G12.01*)*/AT2G06210
CCATATCTGCAATCGCGTTTTCGCG	Genotyping (ID 2333)	T-DNA insertion *cdc73-1* (SALK_150644C)/AT3G22590
ATGACTATTTTGCCAATACACCC	Genotyping (ID 2332)	T-DNA insertion *cdc73-2* (SALK_008357)/AT3G22590
GGTAACATTGTGCTCAGTGGTGG	qRT-PCR (ID 1312)	AT3G18780
GGTGCAACGACCTTAATCTTCAT	qRT-PCR (ID 1313)	AT3G18780
TGGGAAAGTGTTGCCATCC	qRT-PCR (ID 4973)	AT1G13440
CTTCATTTTGCCTTCAGATTCCTC	qRT-PCR (ID 4974)	AT1G13440
CTCACATTTTCGTAGCCGCAAGAC	qRT-PCR (ID 3394)	AT1G07940
GATCAAGTGACCAGTTGTGGTCGAT	qRT-PCR (ID 3395)	AT1G07940
TCCAAGTCTAGCCTCTGGCCTTC	qRT-PCR (ID 1825)	AT5G59310
CTTCACTTGATGGTGGCGCAGT	qRT-PCR (ID 1826)	AT5G59310

Table S2. Summary of the RNA-seq data

Samplename	Sequenced reads	Discarded during QC	Uniquely mapped	Multimapped	Remaining after Deduplication	intron/exon overlapping reads
cdc73-2_control_R1	40.957.291	154.997	28.235.140	12.147.390	8.914.355	6,3%
cdc73-2_control_R2	41.322.046	124.714	14.627.096	23.585.783	7.800.016	5,0%
cdc73-2_control_R3	36.115.587	179.703	24.035.484	11.717.798	10.764.356	6,6%
cdc73-2_control_R4	34.135.122	152.327	22.183.405	11.599.793	7.887.476	6,2%
cdc73-2_salt_R1	45.431.190	171.662	32.391.349	12.491.613	14.760.038	6,6%
cdc73-2_salt_R2	47.841.610	221.609	29.509.373	17.811.550	9.703.744	5,9%
cdc73-2_salt_R3	56.034.250	305.620	34.307.556	21.215.494	14.302.596	5,2%
cdc73-2_salt_R4	31.014.258	136.776	15.769.109	14.989.041	6.181.114	5,0%
Col-0_control_R1	52.500.916	242.370	33.862.128	18.026.898	16.172.392	6,4%
Col-0_control_R2	42.852.940	154.600	28.556.096	13.952.985	13.180.075	5,9%
Col-0_control_R3	47.132.808	228.127	28.988.654	17.700.578	11.463.268	5,3%
Col-0_control_R4	32.791.473	163.168	20.995.244	11.479.085	8.409.818	5,3%
Col-0_salt_R1	45.852.000	169.525	31.767.808	13.652.069	11.853.097	6,3%
Col-0_salt_R2	47.406.912	166.789	30.058.373	11.591.679	15.240.906	6,5%
Col-0_salt_R3	29.713.152	132.703	20.798.850	8.651.038	8.876.253	5,2%
Col-0_salt_R4	47.891.923	165.539	34.704.628	12.850.296	13.463.462	5,3%
elf7-3_control_R1	38.198.310	166.468	28.480.918	9.329.676	14.423.342	6,3%
elf7-3_control_R2	43.147.065	173.413	29.999.234	12.613.487	13.903.721	6,4%
elf7-3_control_R3	51.036.832	180.524	36.620.562	13.791.698	17.086.004	5,4%
elf7-3_control_R4	34.714.729	151.728	25.457.460	8.935.111	9.971.259	5,5%
elf7-3_salt_R2	36.608.939	175.902	25.649.831	10.510.374	13.563.965	6,0%
elf7-3_salt_R3	43.886.319	146.100	25.634.545	17.930.644	12.739.279	5,1%
elf7-3_salt_R4	45.686.898	154.693	24.634.379	20.769.580	10.033.202	5,0%
